# A Bespoke Social Network for Deaf Women in Ecuador to Access Information on Sexual and Reproductive Health

**DOI:** 10.3390/ijerph16203962

**Published:** 2019-10-17

**Authors:** Yaroslava Robles-Bykbaev, Christian Oyola-Flores, Vladimir Espartaco Robles-Bykbaev, Martín López-Nores, Paola Ingavélez-Guerra, José Juan Pazos-Arias, Fernando Pesántez-Avilés, Manuel Ramos-Cabrer

**Affiliations:** 1 GI-IATa, UNESCO Chair on Support Technologies for Educational Inclusion, Universidad Politécnica Salesiana, 010102 Cuenca, Ecuador; zrobles@ups.edu.ec (Y.R.-B.); coyola@est.ups.edu.ec (C.O.-F.); pcingavelez@ups.edu.ec (P.I.-G.); fpesantez@ups.edu.ec (F.P.-A.); 2 AtlantTIC Research Center, Department of Telematics Engineering, University of Vigo, 36310 Vigo, Spain; mlnores@det.uvigo.es (M.L.-N.); jose@det.uvigo.es (J.J.P.-A.); mramos@det.uvigo.es (M.R.-C.)

**Keywords:** Sexual and Reproductive Health, deaf women, Ecuadorian Sign Language, hearing loss, recommender system

## Abstract

Many deaf women face the lack of numerous resources related to their personal development. The unavailability of proper information on Sexual and Reproductive Health (SRH), in particular, causes problems of sexually transmitted infections, unwanted pregnancy in adolescence, sexual violence, complications during pregnancy, etc. In response to this, we have created a social network that delivers SRH content (verified and validated by experts) to women with different degrees of hearing loss. The site features a recommender system that selects the most relevant pieces of content to deliver to each woman, driven by her individual preferences, needs and levels of knowledge on the different subjects. We report experiments conducted in Cuenca, Ecuador, between 2017 and 2018 with 98 volunteers from low- and middle-income settings, aiming to evaluate the quality and appeal of the contents, the coherence of the methodology followed to create them, and the effectiveness of the content recommendations. The positive results encourage the frequent creation of new content and the refinement of the recommendation logic as the cohort of users expands over time.

## 1. Introduction

According to the latest data published by the World Health Organisation, more than 466 million people around the world (over 5% of the world’s population) have disabling hearing loss, the majority living in low- and middle-income countries [1]. This figure includes 34 million children, whose problems often go unnoticed in cases of mild and moderate hearing impairment, leading to cognitive-linguistic shortcomings and severe lags in educational and social development over the years. Thus, on the one hand, many hearing-impaired youngsters who have finished primary school still suffer from functional illiteracy or have serious difficulties in understanding texts and expressing themselves [2,3]. On the other hand, a survey conducted in 2009 [4] showed that most of the low- and middle-income countries fail to cater for those shortcomings, as only 43 (out of 93 respondents) had some kind of sign language training, and 30 of them had 20 or fewer qualified interpreters [4,5]. These facts, altogether, have a significant impact in the health care area, since the communication barriers between patients and caregivers increase the odds for inaccurate diagnoses and treatments [6], whereas the lack of accessible information about healthy lifestyle and disease prevention leads to particularly vulnerable cohorts [7,8]. The literature gathers abundant evidence that deaf people generally suffer from lower health literacy than the overall population [9,10,11].

In this paper, we consider the specific situation of deaf women in Ecuador, in relation to their access to Sexual and Reproductive Health (henceforth, SRH). As of September 2019, there are 30.078 deaf women in the country (0.4% of the female population) and, although Ecuador is among the developing countries that do provide sign language interpretation training and services [5], the *National Council for the Equality of Disabilities* (CONADIS) indicated in its 2017 report that none of the 3.847 healthcare facilities of the country can offer comprehensive support to deaf people yet. Moreover, the illiteracy ratios among deaf people are much greater than among non-deaf or regular people, and the taboo surrounding sexual and reproductive issues contributes to a large knowledge gap between hearing and non-hearing women during their maturation years and ever after those [12]. In consequence, the situation in relation to disability and access to SRH resembles that of countries with scarcer resources per inhabitant [13].

In order to help alleviate this problem, we have created a social network that delivers SRH content, along with information regarding beauty and nutrition, to women with different degrees of hearing loss. We designed the social network following the results of an ethnographic study conducted in 2016 with deaf women from two collaborating associations, which resulted in the definition of guidelines for the creation of appropriate contents. On the technical side, the social network features a content management system that keeps metadata about the contents and the users, so that a recommender system can proactively select the most relevant pieces of content to deliver to each woman upon successive interactions, driven by her individual needs and levels of knowledge on the different subjects.

The paper is organised as follows. In Section 2 we discuss related work focused on delivering accessible health-related material to deaf people. Section 3 describes the main features of our social network, whereas Section 4 presents the results of quantitative studies aimed at assessing the deaf women’s knowledge about SRH, the potential impact of the social network among them, and the value of the content recommendations. Conclusions and future work are presented included in Section 5.

## 2. Overview of Support Systems for SRH

Recent years have witnessed the development of a growing number of online services aimed at delivering information on social, cultural and health-related topics to deaf people. These services are typically bound to a specific geographical area (e.g., a state or a country) due to the fact that sign language varies across different regions (The Ethnologue (https://www.ethnologue.com/subgroups/sign-language) currently lists 144 sign languages). In Spain, for example, the *Andalusian Federation of Deaf People Associations* (FAAS) and the *Spanish Society of Family and Community Medicine* (SEMFYC) worked together to create a web platform hosting videos with advice on pregnancy, baby care, hypertension, vaccines, *…* all captioned in Spanish Sign Language. Such contents constitute the “*Practical Guide to Health*”, which aims to complement, in an accessible way, the information provided in medical consultations to deaf people in Spain [14]. Shortly before, an online program in American Sign Language (meaning the variant used in the USA) allowed different researchers to find evidence that accessible contents helped deaf women to acquire useful and consistent SRH knowledge in relation to ovarian cancer detection, sexually transmitted infections, prenatal controls, etc. [15,16]. By the same time, in France, Legeay and Saillard [17] presented an experiment in which a group of young people participated in the creation of videos with captions in French Sign Language to be posted on a web site. Their active involvement resulted in notable advancements in relation to the acquisition and development of psychosocial skills in health education.

In addition to the creation of accessible contents, we have seen numerous uses of social networks as tools to increase deaf people’s communication. As noted in [18], social networks have made people in general more adept at written and visual communication versus purely verbal communication, causing a huge change in how the deaf (at least, those who have access to written information as well as to the Internet) interact with the rest of the world. Facebook, Twitter, Instagram and other general-purpose sites have even helped to remove stigmas that the deaf were intellectually inferior [19]. These major players, however, still leave place for niche, bespoke social networks hosting contents targeted at specific communities. In this line, it is worth highlighting the project presented in relation to a website called FactNotFiction [20], that offers medically accurate contents for young people who have inadequate access to SRH in the USA state of Mississippi. The authors discuss the improved reach and effectiveness of the social media campaigns driven by *behavioural targeting*. This is an advertising mechanism used to gain better understanding of particular populations by collecting information on their web browsing behaviours, and then placing ads on the websites they visit most frequently [21]. The study also emphasises the fact that the site reached through the ads (in this case, FactNotFiction) should be designed to be dynamic, given that fresh content is integral to retaining youth on social media sites.

Informed by these findings, we have created a bespoke social network for deaf women in Ecuador, which yields guidelines for similar developments in other countries. Besides the specificities of structure and format, our site features a recommender system that addresses (as far as we know, for the first time) a persistent problem in this kind of online resources: it is not enough to attract the target users to a web site; once they have landed on it, it is necessary to face them with properly selected content, in order to make the most of the short time that they are likely to spend browsing its content [22].

## 3. An AI-Enhanced Social Network on SRH for Deaf Women

**Figure 1:**
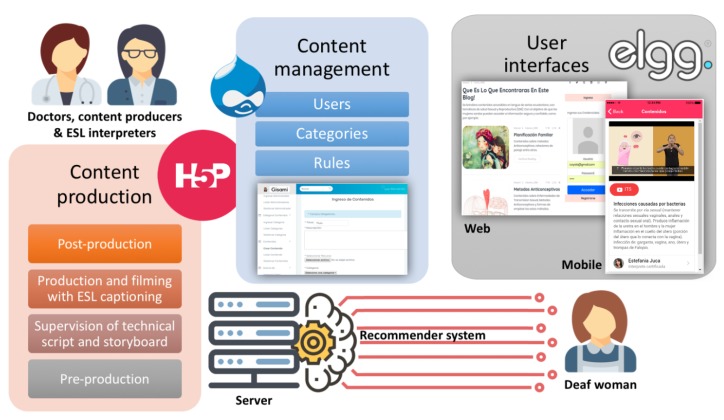
The main parts of our social network.

The diagram of Figure 1 shows the main parts of our bespoke social network for deaf women. The target user is represented on the right hand side, as she can interact with the site and its contents via a web browser or a mobile app. On the left hand side, in turn, we represent the doctors, content producers, educators and sign language interpreters who work together in content design and preparation. The following subsections present the most relevant aspects of (i) the guidelines we designed to conduct the content creation processes, plus our first experience with them, (ii) the content management system and its key elements of information, and (iii) the mobile and web user interfaces.

### 3.1. Content Creation Guidelines

Grounded on a review of the literature in relation to the creation of accessible multimedia contents for hearing-impaired people [23,24], accumulated evidence on how to design and deliver SHR information [25,26] and experiences in running bespoke social networks targeted at certain social groups [27,28], we carried out an ethnographic study in 2016 to involve deaf women in the identification of guidelines for the creation of new materials to offer in our social network.

In this study, we took a qualitative approach as recommended in [29] to understand the deaf women’s views towards education and health-related information. We enjoyed the collaboration of two deaf women who served as key informants and representatives of the National Federation of Deaf People in Ecuador and the Association of Deaf People from the Azuay province, plus three qualified interpreters of Ecuadorian Sign Language (ESL). With their aid, we designed, implemented and translated semi-structured interviews that we applied later on with other deaf women, both literate and illiterate (the same who participated in the quantitative studies of Section 4). Prior to the interviews, we requested informed consent from each participant, explaining the terms and conditions with the help of ESL interpreters. The procedure was supervised by the Ecuadorian Service of Professional Capacitation, that also cared for ethical approval and confidentiality requirements. At this point, we found it was necessary to add new signs to the Ecuadorian Sign Language Dictionary [30] (see Appendix A) to make sure that the deaf women would freely and voluntarily join the study and provide information about their experiences, knowledge and expectations.

From the analysis of the interviews, we created a guide for content producers and ESL interpreters that covers the following stages:

- Firstly, a pre-production stage must be conducted by a multidisciplinary team of experts from areas of medicine, graphic design, education, social communication, computer science and sign language interpretation. Among other tasks, the team determines the terminology and the interpretation techniques that will be used in the new contents, and designs conceptual and graphic scripts for the subsequent stages.
- Following the pre-production, the team of experts develops storyboard notes for the illustration and animation processes. This task helps to substantiate the scripts, with a twofold objective: on the one hand, it allows supervising the sequencing of the different topics, aiming to ensure that the users will not experience any continuity gaps; on the other hand, it facilitates the identification of a coherent set of visual styles and clues.
- In preparation for production and filming, proper clothing and spaces must be decided for the sign language interpreters. The design and animation process is carried out, and accessible subtitling is added to help deaf women who have post-linguistic hearing loss and are able to read text.
- Finally, the post-production stage is needed to deal with the editing and optimization of graphics and animations. The resulting materials must be evaluated by a group of deaf women, in order to check whether everything is understable for all age ranges. Only the contents that meet the proper quality and understandability levels are uploaded to the social network.

The guide was supplemented with tutorials on the use of H5P (https://h5p.org/), a plugin for publishing systems that makes it easy to create, share and reuse highly interactive web content. Working on the Drupal content management system (https://www.drupal.org), these tutorials empowered the collaborating doctors, content producers and ESL interpreters to create rich materials efficiently, out of a sizeable set of content types and templates. It is important to note that we could not have any native ESL signers do the signing on the videos, owing to the very problem that we were targeting: even the literate deaf women we contacted (including the two key representatives mentioned above) did not understand many of the SRH concepts appearing in the scripts, so they were unable to render them properly. The certified ESL interpreters, in contrast, were fully familiar with the concepts, which they used regularly, both orally and written.

Another point worth highlighting is that the social network delivers not only information about SRH; rather, this is entwined with advice on beauty and nutrition, which are two pillars on which SRH develops as part of physical well-being and self-esteem [31,32]. These additional contents serve a twofold purpose: (i) to increase the allure of the platform as a whole among its target public, so that it is not perceived as a site that simply delivers authority-approved advice, and (ii) to allow offering more dynamic and lively content than would be possible exclusively with SRH information, whose core messages are subject to sporadic updates only.

### 3.2. The Content Management System

**Figure 2:**
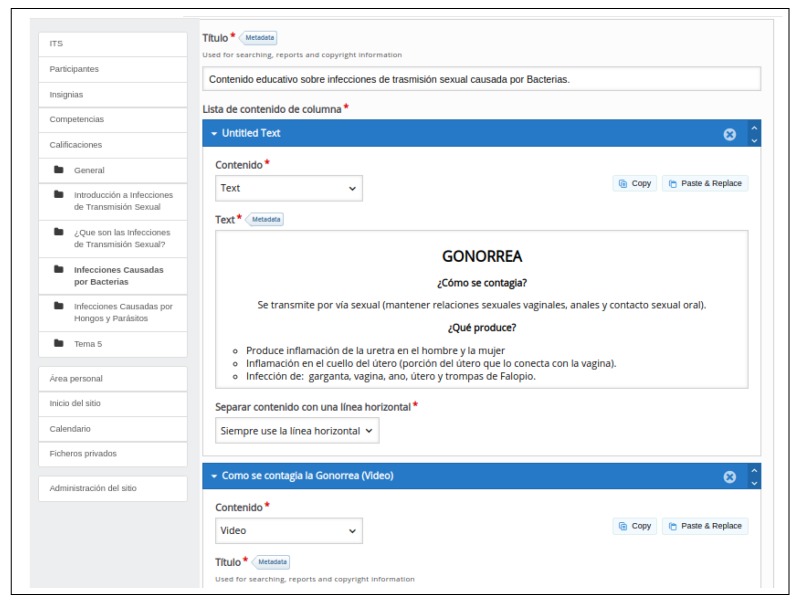
The interface to add a new piece of content and to annotate it with the corresponding categories.

Our content management system is responsible for hosting the information offered within the social site, allowing the content producers to upload, annotate, edit and delete individual assets. In order to cater for our specific needs, we have extended the Drupal platform with plugins of our own, dealing with the following key elements:

- Categories: each piece of content is annotated with a number of categories, which doctors and content producers select from a purpose-made thesaurus that covers all the relevant topics in SRH, with some finer-grained detail for tags related to beauty and nutrition (see Figure 2).
- Users: each user has a profile that keeps track of her interactions with the platform as a whole (e.g., the categories she browses, the queries she types to search for content, etc.) and with each particular piece of content (e.g., her replies to expert-designed questionnaires, how much of a video she has watched, etc.). From the information so gathered, we estimate her levels of knowledge about the different categories.
- Rules: the recommender system that matches users and pieces of content is driven by a set of rules, entered and revised over time by experts. A rule may involve any arithmetic operations on conditions related to (i) the categories linked to each piece of content and (ii) the inclusion of those categories in the user profiles and the corresponding levels of knowledge.

As depicted in Figure 1, contents, categories, user profiles and rules are kept in a server, that is also in charge of executing the logic of the recommender system. The operation of the latter is described in Appendix B.

### 3.3. The User Interfaces

**Figure 3:**
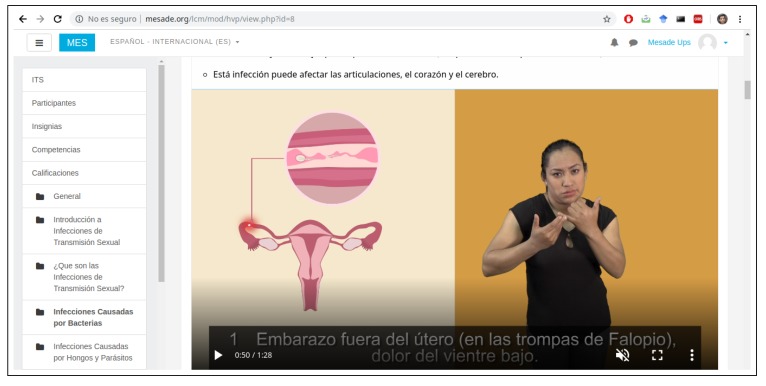
Snapshot of the web version of the user interfaces.

**Figure 4:**
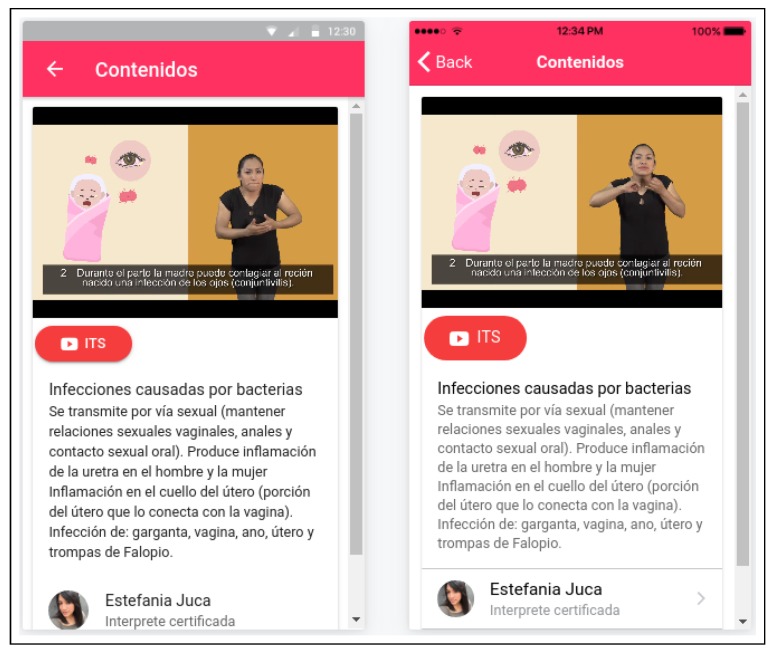
Snapshot of the mobile version of the user interfaces.

We have built the main interfaces offered to the deaf women using Elgg (https://elgg.org), an open-source social networking engine that provides common but highly customisable elements with which to build all kinds of social environments featuring blogs, chats, user groups, etc. Figure 3 shows a snapshot of the web version, with the login form and a non-personalised display of recently added contents. Figure 4, in turn, shows a snapshot of the mobile version while playing a video about the prevention of sexually transmitted infections.

## 4. Quantitative Evaluation Experiments

In order to have a complete analysis of our proposal between 2016 and 2018, we conducted an evaluation in the city of Cuenca, consisting of three experiments:

- In the first one, in parallel with the ethnographic study of Section 3.1, we worked with 31 deaf women to assess their knowledge about the most relevant SRH topics.
- After the design and development of the social network and the creation of the first set of contents were complete (mid 2017), we offered them to a new group of 26 deaf women and three experts for evaluation.
- Finally, during the first semester of 2018, we assessed the value of the recommender system with the aid of 22 students of a Bachelor Degree in Initial Education and 16 students of a Master’s Degree in Special Education.

These experiments shared the same ethics procedures described in Section 3.1, under the supervision of the Ecuadorian Service of Professional Capacitation. The data were handled by descriptive statistics using the R software, mainly looking at measurements of central trends, frequencies and percentages.

### 4.1. Deaf Women’s Knowledge about SRH

As a previous step to design and develop a novel educational tool to teach SRH concepts to deaf women, we wanted to assess their knowledge about three pillars of SRH, namely prenatal care, family planning methods and STIs. To this aim, with the aid of doctors and SRH experts, we ran individual interviews with 31 volunteers:

- Fifteen women came from the National Federation of Deaf People in Ecuador and the Association of Deaf People from the Azuay province—the contacts were facilitated by the two key informants and representatives who had participated in the ethnographic study of Section 3.1.
- Thirteen women came from the Febres Cordero high school and the Dolores J. Torres high school, with which our institution (Universidad Politécnica Salesiana) had signed formal agreements for collaboration in areas of inclusive education.
- Three women came from the Jehovah’s Witnesses Congregation of Cuenca, that was reached through systematic searches on the Internet.

These women made up a diverse population, with ages ranging from 17 to 53 and monthly earnings from USD 0 to 817. One woman had no formal education, whereas seven had finished primary school, 19 had finished high school and four were at bachelor level. Sixteen lived in rural areas and 15 in urban ones; 22 were single, four were married, four were divorced and one was living in free union.

The scale used for the evaluation had three levels: low (1–4 points), medium (5–9) and high (10–15). However, we found that only four women (roughly 13%) reached the medium level, and no one with averages greater than 6. Notwithstanding the small sample of users, these data reinforced our original motivation in line with the literature review, showing that deaf women are a highly vulnerable population with many difficulties to access education and healthcare and, in general, to lead a full life within the culture of non-deaf or regular people. The study was validated using Cronbach’s Alpha test [33], which yielded a value of 0.82.

### 4.2. Deaf Women’s Perceptions about the Social Network

Once we had created the social network and it was online with a first set of contents, we wanted to assess the value perceived by deaf women and experts. Thus, on the one hand, we resorted to convenience sampling to involve 26 deaf women (different from the preceding study) from the Association of Deaf People from the Azuay province (14), the Febres Cordero high school (8) and the Dolores J. Torres high school (4), their ages ranging between 18 and 50. On the other hand, we enjoyed the collaboration of three ESL interpreters and caregivers from the Association of Deaf People from the Azuay province.

After one week with access to the platform, the deaf women had to complete a survey that we designed along with physicians, sign language interpreters and an anthropologist. The survey contained a number of items that the deaf women had to rate on a 5-point Likert scale: 1 meaning “most negative” and 5 meaning “most positive” [34].

**Figure 5:**
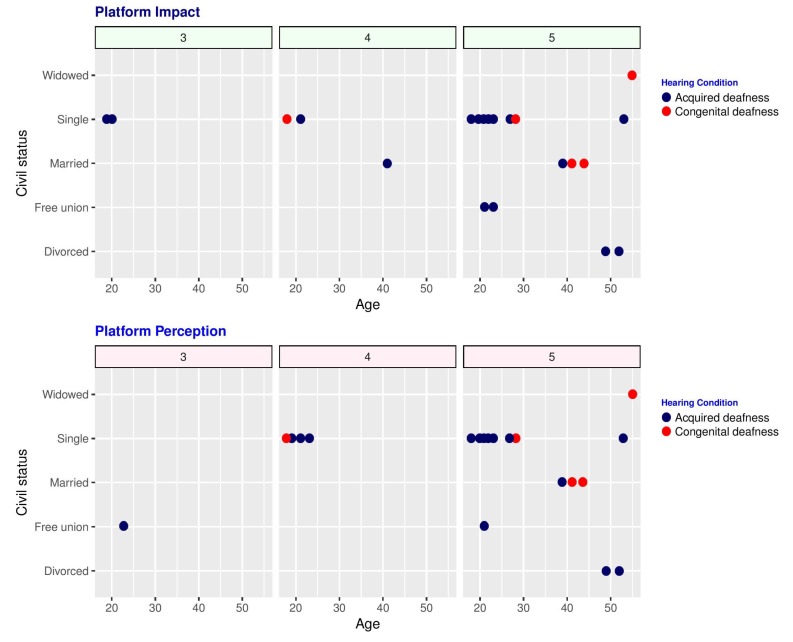
Summary of the deaf women’s ratings for potential impact and perception of the social network.

Figure 5 graphs the ratings gathered for the potential impact of the platform within the deaf community, as well as the overall perception, differentiating women with congenital or acquired deafness by the color of the dots. It can be seen that there was a significant majority of five ratings, and no rating below 3. Again, the study was validated using Cronbach’s Alpha test, which yielded a value of 0.85. Further analysis allowed to ascertain that the positive perception of the platform is independent of the women’s instruction level.

In turn, the experts and caregivers were asked to rate, on a 10-point scale, the parameters defined in the first column of Table 1. Possibly motivated by the previous absence of comparable solutions, the experts enthusiastically evaluated the social network as an attractive and engaging product, suitable and accessible to all deaf women in Ecuador, and with excellent features to support the learning process. Yet, they were dubious about the self-learning aspect, arguing that some expert guidance and group activities can reinforce the user’s willingness to fully watch new videos and do the assessment activities, whereas leaving her alone might eventually lead to superficial watching and no interest for the self-evaluation. In the same line, they were concerned about the poorest and the illiterate, who could hardly use these services unless in communal settings. Finally, regarding the applicability to promote knowledge about SRH in Ecuador, the experts wondered about the need for institutional support to advertise the platform thoroughly and to ensure steady creation of new materials.

### 4.3. Evaluation of the Recommender System

In the final study, we evaluated the recommender system with the collaboration of 22 students of a Bachelor Degree in Initial Education and 16 students of a Master’s Degree in Special Education, all female and approached by convenience sampling in the Cuenca Campus of Universidad Politécnica Salesiana. This group of volunteers provided us with valuable feedback from the viewpoints of future professionals who will work with children and youth, as well as the critical criteria of experts who already work with people with disabilities.

**Figure 6:**
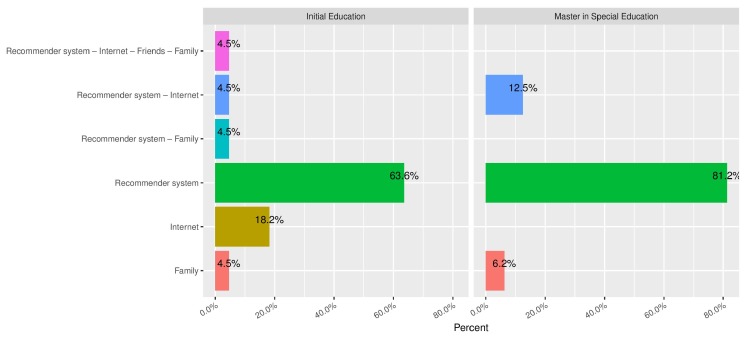
The preferred sources for learning SRH concepts.

The volunteers filled in a questionnare that we designed along with physicians and ICT experts. Figure 6 shows one of the major highlights, with the responses to the question of which sources the volunteers thought could make it easier for deaf women to properly learn about SRH. They could tick several options among “Internet” (general sites), “family”, “friends” and our recommender system. The latter was chosen by 81.2% of the Bachelor Degree students and 63.6% of the Master’s Degree students, which is clearly a sign that this technological solution helps to fill in a persistent gap.

**Figure 7:**
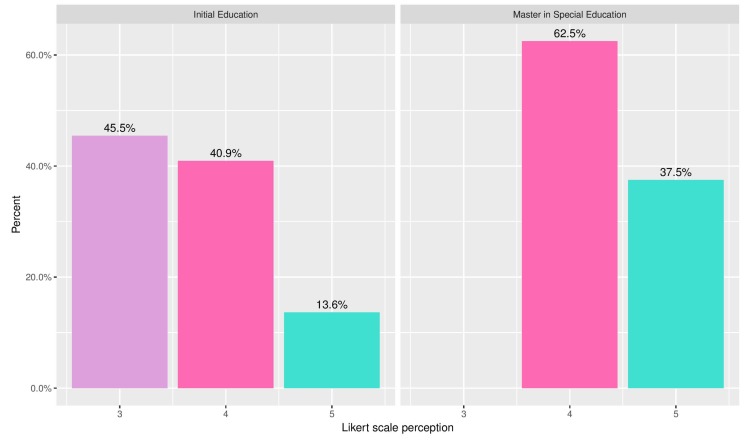
The pertinence of contents selected by the recommender system.

**Figure 8:**
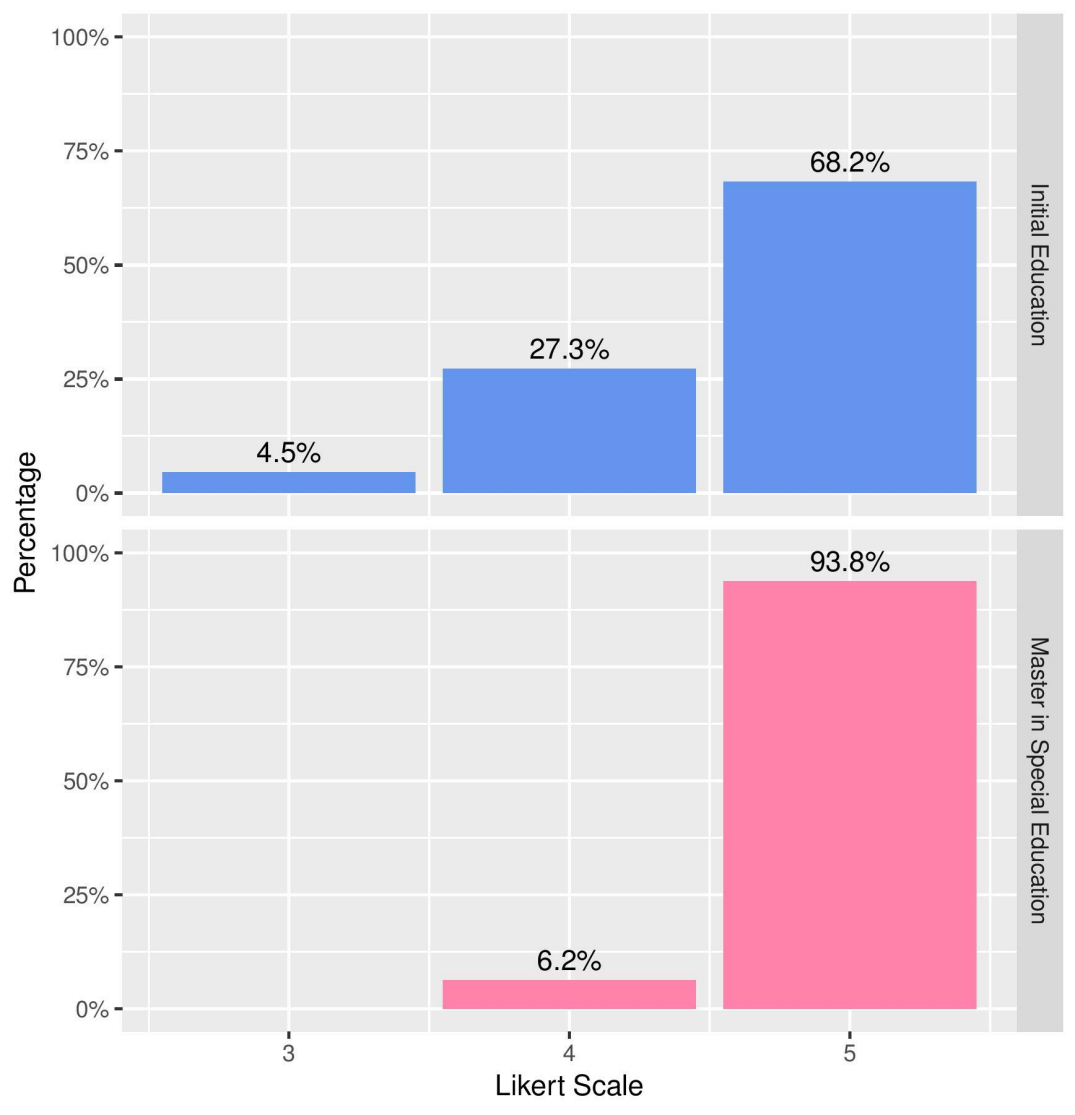
The importance of implementing the recommender system in educational institutions.

Figure 7 shows the ratings (on a 5-point Likert scale) provided by the volunteers when asked about the quality of the personalised recommendations of content, based on levels of knowledge. It can be seen that both groups considered the offered contents appropriate; noticeably, however, the students of the Master’s Degree in Special Education provided ratings with a statistically significant higher average, due to their awareness of the challenges faced by the deaf community. In the same line, Figure 8 shows the replies gathered in response to the question of how important each volunteer thought it would be to offer the contents selected by the recommender system as part of the official education plans throughout the country.

This survey was validated using Cronbach’s Alpha test, which yielded a value of 0.85.

## 5. Conclusions and Future Work

Deaf women are a highly vulnerable cohort, facing considerable challenges in education and healthcare, which prevent them from fully developing their capabilities as humans. Access to information about sexual and reproductive health is a particularly important gap, since most of the initiatives developed in the past have left deaf people aside. This has been shown to cause a noticeable disadvantage that eventually leads to lack of control over family growth, more limited opportunities in the labour market and worse quality of life.

The massive use of online social networks has created opportunities for deaf people to engage more actively with society. Following up on previous works that sought to deliver SRH content to this community, we created a bespoke social network that, for the first time, deals with the problem of selecting pieces of content proactively in order to maximise the learning outcomes. Our platform (available at http://mesade.org/mcs/) has been positively perceived by a sample population of deaf women from Ecuador, with diverse demographic, educational and health-related conditions. Sign language interpreters, educators and caregivers have given very positive ratings, too, for the overall design of the social network, the support it provides for learning about SRH, the guidelines for the creation of more accessible contents, the quality of the content recommendations and the interest of integrating these solutions in educational settings. Notwithstanding the small samples of users, the quantitative studies have been validated in relation to statistical significance.

The feedback that we continue to gather from women who use the social network, either alone or assisted by a caregiver, at home or in communal settings, remains positive as new institutions and associations spread the word about it in Ecuador. Therefore, we believe this work can inform ongoing initiatives for policy, practice and research in what concerns the guidelines for the creation of SRH contents for deaf women. One crucial aspect, in our experience, relates to the lack we noticed in Ecuadorian Sign Language of signs to convey concepts about sexual and reproductive health, as well as ethical issues. This lack is probably present in many other sign languages all over the world, acting as a barrier that takes much effort and time to supress.

Anyways, backed by the positive findings, the UNESCO Chair on Support Technologies for Educational Inclusion is working to promote further adoption of our social network throughout Ecuador in a first stage, and Latin America afterwards. As a crucial part of this initiative, we are defining the license terms and agreements to encourage new organisations and institutions to contribute new contents regularly. Finally, on the research side, we are working to define experimental procedures with which to evaluate the effectiveness of the social network as an intervention tool, measuring levels of SRH knowledge over periods of weeks or months accessing its contents. Technically, we are working to create intelligent and personalised avatars that could work as sign language interpreters, aiming to supplement the very scarce human resources. A description of our early developments in this regard can be found at [35].

## Figures and Tables

**Table 1:** Ratings provided by the three experts who evaluated the platform.

Parameter	Assessment			Average
	Expert 1	Expert 2	Expert 3	
Applicability: to what extent the platform can be realistically used to promote knowledge about SRH in Ecuadorian Sign Language?	6	8	7	7
Learning support: to what extent the platform design and features such as tutoring facilities, the assessment quizzes and the recommender system can effectively foster SRH knowledge acquisition?	10	10	10	10
Self-Learning: to what extent the platform can become a self-education environment?	5	5	5	5
Attractiveness: to what extent the platform can keep the deaf women engaged and with motivation to learn about SRH?	10	10	10	10
Suitability: to what extent the available contents are suitable for the whole spectrum of deaf women?	10	10	10	10

**Figure A1:**
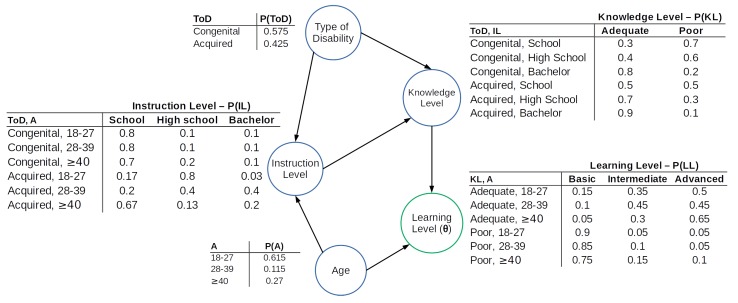
The PGM used to fight the cold-start problem, with probabilities initialised from the stats of the group of deaf women who participated in our experiments.

**Figure A2:**
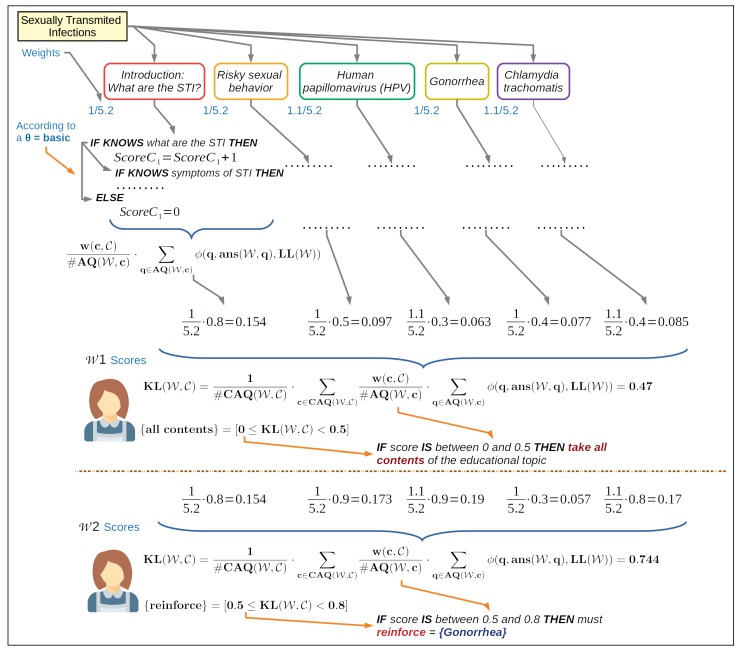
A sample of applying a rule to update knowledge values on the category of STIs.

## Abbreviations

The following abbreviations are used in this manuscript:

AI	Artificial Intelligence
ESL	Ecuadorian Sign Language
ICT	Information & Communication Technologies
SRH	Sexual & Reproductive Health
STI	Sexually Transmitted Infections

## Appendix A. New Terms Added to the Ecuadorian Sign Language Dictionary

The following are some of the words about informed consent and sexually transmitted infections that had to be configured, illustrated, animated, narrated and described by subtitles in videos aimed at deaf women, as they did not exist in ESL:

- Consent
- Privacy
- Confidentiality
- Promiscuity
- Prejudices
- Colic
- Burning
- Secretions
- Ectopic pregnancy
- Lymph nodes
- Fungi
- Parasites
- Microorganisms
- Bacteria
- Vulva
- Serious congenital malformations
- Sexual transmission
- Anal, oral, vaginal sexual contagion
- Vaccines
- HIV
- Fetid
- Injury

The process of extending a sign language with new terms is very complex, necessarily involving deaf native signers, domain experts and standardization bodies. Our experience, which enjoyed the participation of one anthropologist, shows that the deaf native signers are particularly important actors, as their intuition is the key to make sure that the new signs will be easily learnt and assimilated by deaf people who see them for the first time.

## Appendix B. The Recommender System

Our recommender system is based on computational techniques of Probabilistic Graphical Models (hereafter, PGMs) and rule-based reasoning. This approach was chosen because we are continuously collecting data to update the deaf women profiles and, besides, the recommendations must evolve as new contents are uploaded to the social network. The system was implemented in Python, using the “Simple Python RecommendatIon System Engine” scikit (SurPRISE) and linking to the R software for statistical computations.

The PGM is intended to avoid the *cold start* problem, i.e., the poor recommendations that derive from the lack of knowledge about new users [36]. As can be seen in Figure A1, it models the causal relationships between the 5 key variables —identified in a focus group with deaf women, interpreters and doctors— as the main aspects of the SRH educational process and the user profiles:

- The *type of disability* (ToD) variable represents the hearing loss diagnosis. We found it sufficient to differentiate only two options: congenital (the woman was born with deafness) and acquired (the woman lost her auditory skills due to an accident or a disease).
  The probability that a randomly chosen deaf woman has congenital deafness is determined by Equation (A1):
  (A1) $$p\left(ToD=congenital\right)=\sum_{I\;\in \;AB50}\frac{women\;with\;impairment\;I}{total\;women\;with\;congenital\;deafness}$$
  where AB50={AB50.0,AB50.1,…} represents the set of congenital hearing impairments defined by the International Classification of Diseases (ICD) [37]. For example, AB50.0 is the code for “*Congenital conductive hearing loss*”. In turn, the probability of acquired deafness is obviously computed as p(ToD=acquired)=1−p(ToD=congenital).
- A woman’s *age* (*A*) is strongly related to the contents that must be covered during the learning of SRH concepts. As above, we derive the probabilities corresponding to the different age ranges by counting how many deaf women fall in each range and dividing by the total number. The age table in Figure A1 does not have a category for women younger than 18 because we did not involve minors in our experiments of delivering SRH content.
- We used Equation (A2) to derive the conditional probabilities of the different *instruction levels* (IL), which in our experiments came down to 3 classes: primary school, secondary school or university. The probabilities are conditional to type of disability and age, so P(il|tod,a) is the *a priori* probability that a deaf woman reached instruction level il given that she has type of disability tod and age *a* (In Equation (A2) it is assumed that the type of disability tod does not depend on age *a*, hence P(tod|a)=P(tod)):
  (A2) $$\begin{array}{cc} P(il,tod,a) & =P(il|tod,a)\cdot P(tod|a)\cdot P(a)\\ \; & =P(il|tod,a)\cdot P(tod)\cdot P(a) \end{array}$$
- By *knowledge levels* (KL) we represent a deaf woman’s overall apprehension of SRH topics, simply differentiating *average* from *poor*. Just like above, we used Equation (A3) to derive the probabilities for the PGM, where P(kl|tod,il) denotes the *a priori* probability that a woman reached knowledge level kl given that she has type of disability tod and instruction level il.
  (A3) P(kl,tod,il)=P(kl|tod,il)·P(tod|il)·P(il)
- From the preceding variables, the PGM can determine which *learning level* (LL) should be used for a deaf woman for whom we do not have precise observations yet. To this aim, we use Equation (A4), where P(ll|kl,a) denotes the *a priori* probability of choosing learning level ll for a woman who has instruction level il and age *a* (In Equation (A4) it is assumed that the knowledge level kl does not depend on age *a*, hence P(kl|a)=P(kl). These and all other assumptions were proven valid on all our datasets by the *Belief and Decision Networks* tool provided by AIspace [38]):
  (A4) $$\begin{array}{cc} P(ll,kl,a) & =P(ll|kl,a)\cdot P(kl|a)\cdot P(a)\\ \; & =P(ll|kl,a)\cdot P(kl)\cdot P(a) \end{array}$$

Following the initialization of a new profile according to the PGM and any given information about the deaf woman, the recommendations for her will include contents corresponding to the categories for which her presumed level of knowledge falls under an experts-defined threshold (denoted by μ). Thereafter, her profile will be updated following her interactions with the platform, in order to maintain more precise levels of knowledge, one per category of content (remember Section 3.2). At that point, the recommended contents will also include materials tagged with the categories about which the user has the lowest knowledge levels, regardless of the thresholds. Next, we will explain the way the knowledge levels are updated by rule-based reasoning, driven by the user’s replies to questionnaires.

Figure A2 depicts the computation of updated KL values for the category of sexually transmitted infections (STIs) for two women ($W_{1}$ and $W_{2}$) who have answered to the questionnaires linked to 5 different pieces of content. Two of those questions were rated by the experts as 10% more important than the others, so their weights appear to be 1.1 instead of 1. For each subject’s level of learning LL (basic, intermediate or advanced), the if…then…else… expressions defined in the rules compute individual scores that are weighed into the new KL values, which in this case are considered low for $W_{1}$ (so she should be faced with all STI-related contents) and medium for $W_{2}$ (so she only needs to reinforce the topic of “Gonorrhea”). The weighing is given in general by Equation (A5):

(A5) $$\begin{array}{c} KL\left(W,C\right)=\frac{1}{\#CAQ(W,C)}\cdot \sum_{c\in CAQ(W,C)}\frac{w(c,C)}{\#AQ(W,c)}\cdot \sum_{q\in AQ(W,c)}\phi \left(q,ans\left(W,q\right),LL\left(W\right)\right) \end{array}$$

where:

- KL(W,C) is the new knowledge level for user W about category C.
- CAQ(W,C) is the set of contents linked to category C and to questionnaires that user W has answered to. #CAQ(W,C) is the cardinality of that set, i.e., the number of contents in it.
- w(c,C) is the weight of a piece of content *c* in relation to the category C.
- AQ(W,c) is the set of questionnaires linked to content *c* that user W has replied to. #AQ(W,c) is the cardinality of that set.
- ϕ(q,ans(W,q),LL(W)) is a function that, given a questionnaire *q*, the set of answers provided to it by a given user (ans(W,q)) and the learning level of that user, returns a mark for the questionnaire by checking the applicability of the rules and performing the corresponding computations.
